# Functional Annotation and Comparative Analysis of Cytochrome P450 Protein Family Genes in Nine Chironomidae Species

**DOI:** 10.3390/biology14091111

**Published:** 2025-08-22

**Authors:** Wenbin Liu, Anmo Zhou, Jiaxin Nie, Ziming Shao, Zhe Nie, Yajin Zhang, Chunmian Liu, Chuncai Yan, Shaobo Gao, Yiwen Wang

**Affiliations:** 1 College of Life Sciences, Tianjin Normal University, Tianjin 300387, China; skylwb@tjnu.edu.cn (W.L.);; 2 Grassland Research Institute of Chinese Academy of Agricultural Sciences, Hohhot 010010, Chinagaoshaobo@caas.cn (S.G.); 3 School of Pharmaceutical Science and Technology, Tianjin University, Tianjin 300072, China; 4 Shanxi Key Laboratory of Nucleic Acid Biopesticides, Shanxi University, Taiyuan 237016, China

**Keywords:** cytochrome P450 monooxygenases, Chironomidae species, gene annotation, environmental adaptability

## Abstract

**Simple Summary:**

Insects rely on cytochrome P450s to break down harmful substances and produce essential molecules for survival. While P450s have been studied in many insects, little is known about them in Chironomidae species. This study identified 577 P450 genes across eight Chironomidae species, categorizing them into four clans. By comparing these genes to those in *Drosophila melanogaster*, research found that two clans remained largely unchanged, while two others expanded significantly, likely helping chironomids adapt to varied habitats. Most P450 enzymes in these chironomids are highly similar to their counterparts in fruit flies, suggesting potential functional parallels between the two. These findings deepen our understanding of how chironomids evolve and cope with environmental challenges, offering insights that could inform pest control and pollution management.

**Abstract:**

Cytochrome P450 monooxygenases (P450s) are one of the most widespread enzyme superfamilies in the biological world. In insects, P450 proteins play a crucial role in the synthesis of endogenous substances and the metabolism of xenobiotics. To date, extensive research has been conducted on P450 gene-mediated detoxification and metabolism across multiple insect species. While Chironomidae species—dominant benthic organisms inhabiting diverse ecological niches and playing critical ecological roles—remain largely uncharacterized in terms of P450 protein annotation, with the exception of a single study on *Propsilocerus akamusi*. In this study, we expanded the annotation scope by identifying the P450 protein genes in eight additional Chironomidae species. A total of 577 P450 protein genes were annotated across the eight species, which could be classified into the following four distinct clans: 50 belonging to the CYP2 clan, 258 to the CYP3 clan, 198 to the CYP4 clan, and 71 to the Mito clan. Phylogenetic analysis using *Drosophila melanogaster* as an outgroup revealed that the CYP2 clan and the Mito clan are highly conserved during evolution, while the CYP3 clan and the CYP4 clan have undergone significant expansion. Most P450 proteins in Chironomidae species exhibit clear orthologous relationships with their *D. melanogaster* counterparts. Our research contributes to a better understanding of the evolutionary processes and the physiological functions of P450 proteins in Chironomidae species and lays the foundation for elucidating the role of P450 in environmental adaptability among the Chironomidae species inhabiting diverse habitats.

## 1. Introduction

Cytochrome P450 monooxygenases (P450s) represent one of the most ancient, complex, and vast enzyme superfamilies. They are involved in numerous metabolic pathways and are present in nearly all forms of life, ranging from bacteria to protists, plants, fungi, and animals [1,2]. They have even been identified in certain viruses [3]. The classification of P450s is based on amino acid sequence identity, e.g., members with >40% identity belong to the same family, and those with >55% identity are assigned to the same subfamily [4]. According to the nomenclature of P450s, the first number after “CYP” denotes the family, the subsequent letter indicates the subfamily, and the number following the subfamily designates the individual gene [5]. Given the proliferation of sequences and the resultant expansion in CYP families, a higher-tier classification system, designated as the “CYP clan,” has been implemented [6]. Insect CYP genes are classified into six clans based on their phylogenetic relationships: CYP2, CYP3, CYP4, CYP16, CYP20, and Mitochondrial (Mito) [7]. Sequences that belong to the Mito clan are thought to be situated on the inner mitochondrial membrane; hence, this clan has been named the mitochondrial clan. The CYP16 and CYP20 clans are newly discovered insect CYP clans found in certain species of Apterygota and Paleoptera, and their functions in insects remain unknown [7]. The other four CYP clans have been previously identified and extensively studied.

Insects’ radiation into diverse habitats and food sources has substantially heightened their exposure to toxic or life-threatening environments. Insect P450s play a crucial role in modulating their adaptive capacity to such varied habitats. Insect P450s primarily serve two functions. The first involves the synthesis of endogenous substances critical for insect growth and development, a role predominantly undertaken by genes from the CYP2 clan and the Mito clan. Most of these genes are conserved in evolution and function, participating in the biosynthesis and metabolism of many bioactive compounds within insects, such as 20-hydroxyecdysone (20E) [8] and juvenile hormone (JH) [9]. They are also involved in the metabolism of fatty acids [10], pheromones, and numerous other signaling molecules crucial for insect communication [11] and defense [12]. The second function focuses on xenobiotic metabolism [2,13]. This function is primarily mediated by genes from the dominant CYP3 clan and CYP4 clan, including those in the CYP4, CYP6, and CYP9 families. These families exhibit remarkable evolutionary and functional diversity, with a large number of genes arising from gene duplication. In most insects, the detoxification of pesticides, plant toxins, and other foreign substances is attributed to these families, which protect insects and facilitate their adaptation to environmental chemical stresses [14,15,16,17].

All P450 enzymes exhibit structural similarities, with the following five conserved motifs: the C-helix motif (WxxxR) located at the C-terminal, followed by the Helix I motif (GxE/DTT/S), the (ExLR) motif and the PERF motif (PxxFxPE/DRF) also situated in the K-helix, and finally the heme-binding motif (PFxxGxRxCxG/A) [1]. Among them, the heme-binding motif is the most conserved part of the protein and is commonly regarded as the characteristic sequence of P450 enzymes, as it contains a cysteine residue involved in the binding of heme iron at the fifth coordination site [2].

The P450 protein families in various insect species have been comprehensively identified and characterized. The number of P450 genes varies significantly across different species. For instance, the human body louse, *Pediculus humanus*, has only 36 P450 genes [18], whereas the Southern house mosquito (*Culex quinquefasciatus*) possesses as many as 204 [19]. Other species have also been systematically identified, such as the fruit fly (*Drosophila melanogaster*, with 90 members) [20], house fly (*Bactrocera dorsalis*, with 101 members) [21], Medfly (*Ceratitis capitata*, with 103 members) [22], *Musca domestica* (146) [23], silkworm (*Bombyx mori*, with 84 members) [24], red flour beetle (*Tribolium castaneum*, with 143 members) [16], African malaria mosquito (*Anopheles gambiae*, with 105 members) [25], and the yellow fever mosquito (*Aedes aegypti*, with 159 members) [26]. P450s are closely associated with insecticide resistance. Numerous studies have aimed to reduce insect resistance by silencing members of the CYP4, CYP6, CYP9, and CYP12 families in insects [27,28,29,30].

Chironomidae, a highly distinctive and widely distributed family within the order Diptera, occupies a pivotal position in ecosystems dominated by benthic organisms across many freshwater and some marine habitats. Given their extensive distribution, large population sizes, and sensitivity to environmental changes, Chironomidae insects are often regarded as effective biological indicators for monitoring water quality and assessing the health status of ecosystems. Chironomidae insects possess an extremely strong environmental adaptability, enabling them to survive and reproduce in a wide range of habitats, from extremely cold to arid and hot environments, and from pristine waters to heavily polluted ones. Consequently, they hold significant value in ecological research and environmental monitoring. Due to their role as biological indicators, a series of toxicological studies on Chironomidae have been conducted to date, particularly focusing on insecticides or pesticides. Among these, studies on the function of P450 genes under various xenobiotic stresses have been carried out in *Chironomus riparius* [31], *Chironomus tentans* [32,33], and *Chironomus kiiensis* [34,35].

Regrettably, although Chironomidae is one of the most significant aquatic insect groups, the systematic identification and annotation of the P450 protein family have been limited to *Propsilcerus akamusi* [36]. In this study, we expanded the annotation of the P450 protein family to include eight additional species within Chironomidae: *Clunio marinus*, *Belgica antarctica*, *Polypedilum vanderplanki*, *Polypedilum pembai*, *Chironomus tepperi*, *Chironomus striatipennis*, *C. riparius* and *C. tentans*. These species encompass four genera and belong to the two major subfamilies of Chironomidae: Chironominae and Orthocladiinae, which collectively account for over 90% of modern Chironomidae species [37].

We believe that the P450 gene family shows significant compositional differences among Chironomidae species inhabiting different habitats and exhibits unique patterns adapted to the characteristics of their respective habitats. This is closely related to their adaptive evolution and detoxification capabilities in response to specific environmental factors in their habitats. Our aim is to conduct a comprehensive identification and phylogenetic analysis of the P450 gene family in Chironomidae, enabling more in-depth and precise gene annotation and functional predictions. This will lay the foundation for future investigations into the role of P450 proteins in the environmental adaptability of different Chironomidae species and the evolutionary studies of P450 proteins in Chironomidae species inhabiting diverse environments.

## 2. Materials and Methods

### 2.1. Gene Annotation

To identify the CYP450 protein genes in eight Chironomidae species, we obtained the P450 protein genes of *D. melanogaster* from the FlyBase website (https://flybase.org/, accessed on 5 February 2025). Previous studies have reported the genomic information of these eight Chironomidae species. We downloaded the proteome data of the annotated genomes of these Chironomidae species from the National Center for Biotechnology Information (NCBI) database [38,39,40,41,42] (Table S1). Using the BLASTP tool in TBtools v2.309 software [43], we employed the P450 amino acid sequences of *D. melanogaster* as query sequences to screen the transcriptome databases of these eight Chironomidae species. With an E-value threshold of 1 × 10^−5^, through Blastp searches, we selected the top three sequences with the highest identity as candidate P450 protein genes for each of the eight Chironomidae species. These candidate genes were further confirmed with the support of the HMMPfam domain of P450s (PF00067, downloaded from http://pfam.xfam.org/, accessed on 8 March 2025). After integrating the sequences obtained by the above methods and removing redundant and incomplete sequences, we finally identified the P450 genes in these eight Chironomidae species. The P450 protein sequences of these eight Chironomidae species are shown in Table S2.

### 2.2. Sequence Alignment, Conserved Motif Visualization

We used DNAMAN software (version 7.212, Lynnon Corp., Quebec, QC, Canada) to perform sequence alignments on the retrieved P450 amino acid sequences from eight Chironomidae species, as well as the P450 amino acid sequences of *D. melanogaster*, *An. gambiae*, and *Ae. aegypti*, respectively. Subsequently, the aligned fasta files were uploaded to WebLogo (http://weblogo.threeplusone.com/create.cgi, accessed on 10 June 2025). Five typical motifs of insect P450s were selected to detect and visualize their conservation patterns, including the heme-binding motif (PFxxGxRxCxG/A), Helix-K (E-x-L-R), Helix-C (W-x-x-x-R), Helix-I (A/G-G-x-D/E-T-T/S), and PERF motif (PxxFxPE/DRF). Finally, Adobe Illustrator CS6 software was employed to edit and visualize the phylogenetic tree.

### 2.3. Classification of P450 Transporter Genes and Phylogenetic Analysis

We aligned the P450 amino acid sequences from eight chironomid species with previously identified P450 sequences of *D. melanogaster*, *An. gambiae*, *Ae. aegypti*, and *P. akamusi*. Sequences with a similarity greater than 40% are classified into the same gene family, while those with a similarity greater than 55% are grouped into the same gene subfamily. For naming, we followed the rules of the P450 Nomenclature Committee [44] and referred to the names of genes that have been clearly named by this committee in *D. melanogaster*, *An. gambiae*, *Ae. aegypti*, and *P. akamusi*. We used the first letters or commonly recognized typical abbreviations of the genus and species names to denote each species: Cm for *C. marinus*, Ba for *B. antarctica*, Pv for *P. vanderplanki*, Pp for *P. pembai*, Cp for *C. tepperi*, Cs for *C. striatipennis*, Cr for *C. riparius*, and Cn for *C. tentans*. When naming each gene, we prefixed the gene name with the protein name from the reference species. For proteins with a similarity lower than 40%, we prefixed the gene name with the name of the clan to which the protein belongs and added a unique numerical identifier to each gene. Details of the 577 amino acid sequences are provided in Table S2. Phylogenetic tree construction followed Dermauw W al.’s method [7]. The CYP sequences were aligned using the web-based MAFFT version 7 [45] with the “G-large–INS–1” parameter set. Subsequently, the aligned sequences were trimmed using trimAl v1.4.1 software [46]. A maximum likelihood (ML) phylogenetic analysis was subsequently performed on the aligned dataset using IQ-TREE v2.2.0.8 [47], employing the LG + I + G4 + F substitution model selected as optimal by ModelTest-NG via the Akaike Information Criterion. The analysis incorporated 3000 ultrafast bootstrap replicates [48] with default settings for bootstrap support correction (-bnni) to minimize overestimation and a UFBoot convergence threshold (-bcor 0.99) specifying the minimum correlation coefficient. Finally, Adobe Illustrator CS6 software was employed to edit and visualize the phylogenetic tree.

## 3. Results

### 3.1. Annotation and Classification of Predicted P450s in Eight Chironomidae Species

In this research, we performed supplementary annotation and classification of P450 genes derived from the genomes of eight Chironomidae species. This was accomplished by aligning their sequences with the previously characterized P450 protein genes in *D. melanogaster* and the P450 genes we had previously annotated in the *P. akamusi* genome, based on sequence similarity. Additionally, we carried out a phylogenetic analysis of the P450 gene family across all nine Chironomidae species, including *P. akamusi*.

A total of 577 P450 genes were identified and classified into the four established P450 clans: 50 genes belonged to the CYP2 clan, 258 to the CYP3 clan, 198 to the CYP4 clan, and 71 to the Mito clan. Each of the eight Chironomidae species’ genomes contained representatives from all four P450 clans. Among these genes, 483 can be further classified into 25 families and 38 subfamilies, while the remaining 94 genes can only be identified as belonging to a certain P450 clan (Table 1).

The number of P450 genes in the Chironomidae species varied, ranging from 43 to 152, with an average count of 71. There is a substantial variation in the number of P450 genes among different species of Diptera insects. For instance, *C. quinquefasciatus* and *Ae. aegypti* possess as many as 204 and 164 P450 genes [19,26], respectively, whereas *An. gambiae* and *D. melanogaster* have only 106 and 83, respectively [20,25]. Even within the same family, Chironomidae, notable differences are observed. Species such as *C. riparius* and *P. vanderplanki* have significantly higher numbers of P450 genes compared to other Chironomidae species, with 152 and 105, respectively. In contrast, the number of P450 genes in other Chironomidae species ranges from 43 to 65.

**Figure 1:**
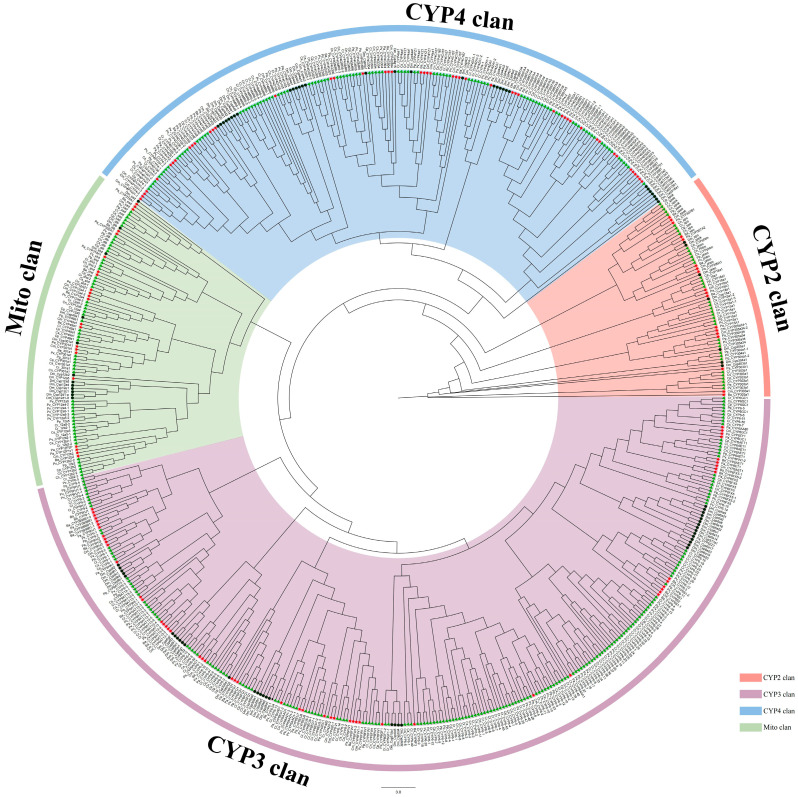
Phylogenetic analysis of P450 protein across nine Chironomidae species and *D. melanogaster*. Black circles represent *D. melanogaster*, green triangles represent Chironominae, red five-pointed stars represent Orthocladiinae. Different clans are marked with distinct colors.

The constructed phylogenetic tree revealed that all the newly discovered P450s could be distinctly classified into the four established clans (Figure 1). To further elucidate the evolutionary placement of P450s within the Chironomidae family, we conducted comprehensive phylogenetic analyses for each clan. The results derived from these analyses are subsequently presented and discussed in the following sections.

### 3.2. Conserved Domain Analysis

**Figure 2:**
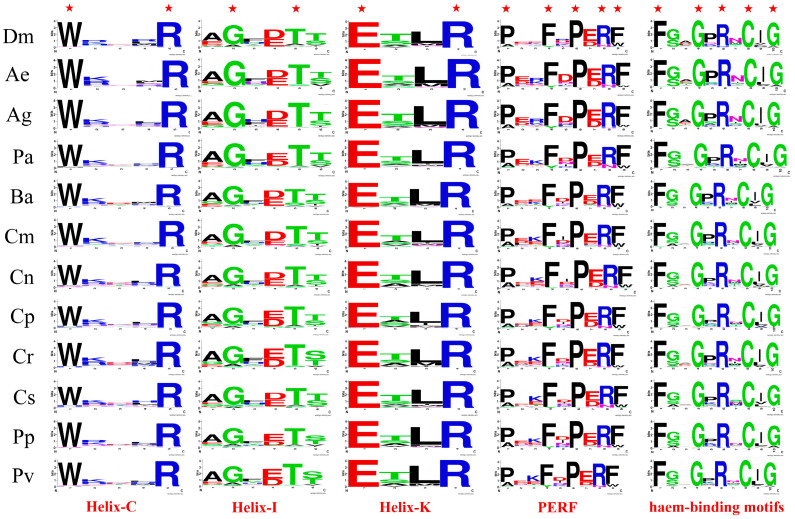
Five conserved motifs of P450 proteins from nine Chironomidae species, *An. Gambiae* (Ag), *Ae. aegypti* (Ae), and *D. melanogaster* (Dm), with red pentagrams indicating the highly conserved amino acid residues within the motifs.

We performed amino acid sequence alignment of P450s from *D. melanogaster*, *An. gambiae*, *Ae. aegypti*, and nine chironomid species. The results showed that the majority of P450 family members possess the five characteristic conserved motifs specific to this family, namely Heme-Loop (F-x-x-G-x-R-x-C-x-G), Helix-K (E-x-L-R), Helix-C (W-x-x-x-R), Helix-I (A/G-G-x-D/E-T-T/S), and PERF (P-x-F-x-P-E/D-R-F) (Figure 2). Consistent with previous research findings, these five conserved motifs in the eight species exhibit a high degree of conservation.

Specifically, the Helix-C (W-x-x-x-R) and Helix-K (E-x-L-R) motifs are highly conserved in *D. melanogaster*, *An. gambiae*, *Ae. aegypti*, and nine chironomid species, especially at amino acid sites crucial for P450 enzyme activity. In the Helix-C motif, amino acids at non-conserved positions show low conservation. The amino acid at the second position of the Helix-K motif is mainly threonine in these 12 species, indicating that the Helix-K motif in Diptera insects primarily follows the ETLR pattern. For the Helix-I motif, similar to *D. melanogaster*, *An. gambiae*, and *Ae. aegypti*, the amino acid at the first position in chironomid species is mainly alanine. At the fourth position, *B. antarctica*, *C. tentans*, and *C. striatipennis*, like *D. melanogaster*, predominantly have Aspartic, while the remaining chironomid species mainly have Glutamic. At the sixth position, except for *C. riparius*, *P. pembai*, and *P. vanderplanki*, which predominantly have Serine, the rest of the chironomid species, like *D. melanogaster*, mainly have Threonine. This suggests that the amino acid at the first position of the Helix-I motif is mainly Alanine in Diptera, while the amino acids at the fourth and sixth positions vary among different chironomid species. Variations in motifs may affect key steps in the catalytic mechanism, such as substrate binding or electron transfer, by altering the enzyme’s structure, thereby influencing its activity or substrate specificity. In the PERF motif, similar to *D. melanogaster*, the amino acids at the first and ninth positions in chironomid species are mainly proline and phenylalanine, indicating that the PERF motif in Diptera may primarily follow the PxxFxPERF pattern. The amino acid at the third position shows low conservation in *D. melanogaster*, but is relatively conserved as Arginine in *An. gambiae* and *Ae. aegypti*, and as Lysine in chironomid species, suggesting that the amino acid at this position may exhibit specificity across different families.

The heme-binding loop harbors the most distinctive consensus sequence (F-x-x-G-x-R-x-C-x-G) characteristic of P450 enzymes. This loop is situated on the proximal side of the heme group, immediately preceding the L helix. Within this sequence, an absolutely conserved cysteine residue acts as the fifth ligand, binding to the heme iron [1]. The cysteine residue within the heme-binding loop is highly conserved among Chironomidae species, *D. melanogaster*, *An. gambiae*, and *Ae. aegypti*. The amino acids at the second and fifth positions of this motif also exhibit relative conservation, predominantly being glycine or serine at the second position and proline at the fifth position. The heme-loop motif in Diptera insects primarily adheres to the F-G/S-x-G-P-R-x-C-x-G pattern. Overall, the high conservation of key amino acids in the five motifs of P450s from Chironomidae species ensures the enzyme’s activity and substrate specificity.

### 3.3. CYP2 Clan

Our previous research demonstrated that members of the CYP2 clan in the genome of *P. akamusi* are distributed across six families (CYP15, CYP18, CYP303, CYP305, CYP306, and CYP307) and seven subfamilies. This study identified 50 new CYP2 clan proteins from eight additional Chironomidae species, classified into seven families and eight subfamilies, including the newly detected CYP304 family, absent in *P. akamusi*.

The CYP2 clan comprises seven family members (15, 18, and 303–307). In most insects, the CYP2 clan is composed of six families. For instance, *B. dorsalis*, *Ceratitis capitate*, and *M. domestica* all lack family 15, while *B. mori* lacks family 304 [21,24]. A small number of insects, such as *T. castaneum* [16], have a CYP2 clan that includes all seven family members. In contrast, the CYP2 clan of *Bemisia tabaci* has significantly fewer family members compared to other insects, consisting of only four families (family 15, 303–305) [49]. Among Diptera, *D. melanogaster* and *An. gambiae* share the characteristic of having six CYP2 clan family members with most insects, lacking family 15 [20]. However, in *C. quinquefasciatus* and *Ae. aegypti*, the CYP2 clan includes all its members, with the number exceeding the seven to nine found in most other insects, having 12 and 11 family members, respectively [19,26]. Among the Chironomidae species, *C. riparius*, *P. vanderplanki*, and *P. akamusi* have six CYP2 clan family members, similar to most insects. *P. pembai*, like *Ae. aegypti*, possesses all the family members of the CYP2 clan. *C. marinus* and *C. tepperi* have five CYP2 clan family members, while *C. striatipennis*, *C. tentans*, and *B. antarctica* have only four CYP2 clan family members, which is significantly fewer than the number of CYP2 clan families in other insects.

**Figure 3:**
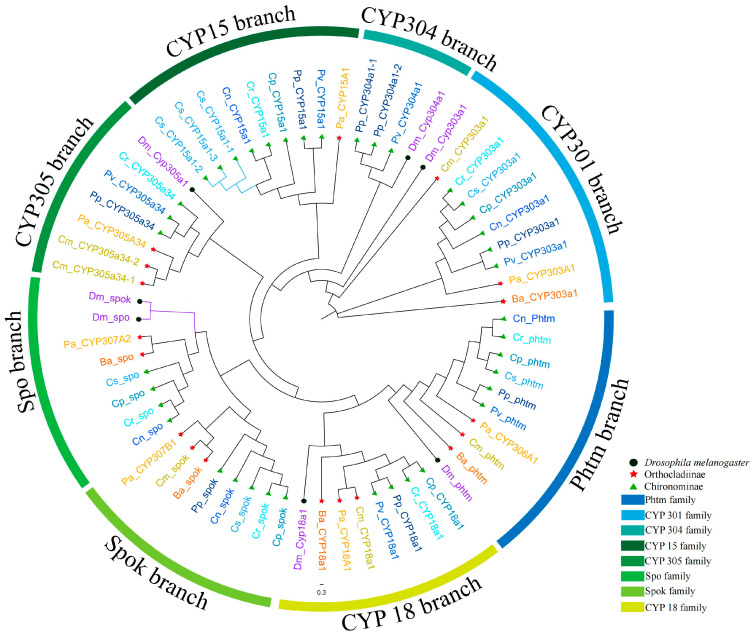
Phylogenetic tree of the CYP2 clan across nine Chironomidae species and Drosophila melanogaster, constructed using IQ-TREE (1000 bootstrap replicates). Black circles indicate CYP2 clan members from *D. melanogaster*. Green triangles represent species from the subfamily Chironominae. Red five-pointed stars denote species from the subfamily Orthocladiinae. Different gene families within the CYP2 clan are distinguished by outer circles of distinct colors. The abbreviations spo, spok, and Phtm correspond to *CYP307A1*, *CYP307A2*, and *CYP306A1*, respectively.

Using the CYP2 clan members of *D. melanogaster* as outgroups, a phylogenetic tree covering all nine Chironomidae species’ CYP2 clans was constructed and analyzed (Figure 3). Interestingly, among the seven CYP2 clan members of *D. melanogaster*, five formed independent branches with their orthologous genes from Chironomidae species in the phylogenetic tree, namely *phtm*, *CYP18A1*, *CYP305A1*, *CYP304A1*, and *CYP303A1*. In contrast, the spo and spok genes clustered separately from the spo and spok branches of Chironomidae. This suggests that the spo and spok genes in Chironomidae originated early in evolution, likely predating the divergence of the Drosophilidae and Chironomidae families. The spo and spok genes in *Drosophila* may have arisen through gene duplication after the formation of the Drosophilidae family. Compared to Drosophila, the Chironomidae family also has a CYP15 branch, which includes one representative gene from each Chironomidae species except for *C. marinus* and *B. antarctica*. Meanwhile, *C. striatipennis* has three genes in the CYP15 branch, likely due to gene duplication. In addition, different Chironomidae species cluster with those in their respective subfamilies, indicating that the CYP2 clan genes of Chironomidae species also exhibit evolutionary conservatism at the subfamily level. Overall, each family of the Chironomidae CYP2 clan formed independent branches in the phylogenetic tree, suggesting a high level of conservation during the evolutionary process of the Chironomidae CYP2 clan. This finding aligns with previous studies on the CYP2 clan family and may be related to the functions of the genes within the CYP2 clan family.

Members of the CYP2 clan are highly conserved both evolutionarily and functionally. Studies have shown that P450 enzymes within the CYP2 family in insects are associated with the biosynthesis or metabolic processes of endogenous compounds, such as *CYP307A1* (*spook*, *spo*), *CYP307A2* (*spookier*, *spok*), and *CYP307B1* (*spookiest*), *CYP306A1* (*phantom*, *phtm*), which are all within the CYP2 clan and belong to a group of genes known as the Halloween genes. These genes are conserved across many insects and are involved in the early steps of the biosynthesis of the insect molting hormone, 20-hydroxyecdysone [17]. *D. melanogaster* possesses two CYP307 genes, namely *CYP307A1* and *CYP307A2*, both of which have been demonstrated to be involved in ecdysone synthesis [50,51,52]. Subsequently, a third paralog, *Cyp307B1*, was discovered in some Coleoptera, Hymenoptera, and certain Diptera species [52]. This paralog originated from an early duplication event that occurred after the divergence of Hymenoptera from Lepidoptera and Diptera species [8]. Given the pivotal role of CYP307 in ecdysteroid synthesis [51,52], it is hypothesized to be involved in the “black box” steps downstream of 7-dehydrocholesterol (7dC). Specifically, it can convert 7dC, which is catalyzed by Neverland (Nvd), into 5β-ketodiol, representing a crucial rate-limiting step in ecdysteroid production [53]. CYP307 exhibits high instability due to its unique structural features and complex catalytic reactions, forming a stark contrast to the conserved nature of other CYP2 clan genes. In our study, we identified two CYP307 genes, *CYP307A2* and *CYP307B1*, in chironomid species, but failed to detect *CYP307B1*. Notably, we did not identify *CYP307A2* in *C. marinus*, *P. pembai*, and *P. vanderplanki*, and *CYP307B1* was absent in *P. vanderplanki*. However, both genes were identified in other chironomid species. This suggests that the *CYP307A1* gene may have undergone lineage-specific loss in chironomid species. The synthesis of 20E in chironomids likely involves the sequential hydroxylation steps mediated by *CYP307A2* and *CYP307B1*. The *CYP306A1* plays a crucial role in ecdysteroid biosynthesis during insect development by hydroxylating ketodiol to convert it into ketotriol in the *B. mori* [54]. We identified the *CYP306A1* gene in all eight chironomid species. Moreover, this gene clusters with its homologous gene in *D. melanogaster* on the phylogenetic tree, indicating that *CYP306A1* in chironomid species is conserved during evolution and may also be involved in 20E synthesis.

The inactivation of 20E is vital for the normal development of insects. CYP18A1 catalyzes the 26-hydroxylation of ecdysteroids and their subsequent oxidation into 26-carboxylic acids. Both loss-of-function mutants of *CYP18A1* and *CYP18A1* RNAi-inactivated strains result in an extended final larval stage and lethal outcomes during development [55]. *CYP18A1* serves as the key enzyme in insects responsible for the degradation of 20E, and its orthologs are present in most insects and arthropods. Apart from *C. striatipennis* and *C. tentans*, we identified orthologous genes of CYP18A1 in several other Chironomidae species. It is possible that chironomid species also degrade 20E through the action of *CYP18A1*. CYP15 is involved in JH synthesis. For example, in the cockroach (*Diploptera punctata*), *CYP15A1* acts as a methyl farnesoate (MF) epoxidase to catalyze JH synthesis. In the *B. mori*, *CYP15C1* acts as a farnesoic acid epoxidase [56], while in the *T. castaneum*, *CYP15A1* exhibits dual farnesoic acid/MF epoxidase activity [57]. Jhamt, as a key methyltransferase, plays a crucial role in the JH synthesis pathway in insects. Specifically, it uses farnesoic acid (FA) or juvenile hormone acid (JHA) as substrates and employs S-adenosylmethionine (SAM) as a methyl donor to methylate these substrates, ultimately generating MF. The activity of Jhamt is precisely regulated by miRNAs, and this regulation further affects JH synthesis [58]. Moreover, the product MF generated by Jhamt is exactly the substrate for CYP15. Whether there are certain microRNAs in insects that can indirectly influence JH synthesis by regulating CYP15 is worthy of in-depth research and exploration in the future. Higher Diptera, such as *D. melanogaster*, lack the CYP15 gene, which may relate to the atypical structure of JH synthesized in their corpora allata (CA). However, CYP15 genes are present in other dipterans, including *Ae. aegypti*, *An. gambiae*, and *C. quinquefasciatus*. Apart from *B. antarctica* and *C. marinus*, we identified the CYP15B1 gene in several other chironomid species, similar to the situation observed in mosquitoes. Their functions remain unknown.

### 3.4. CYP3 Clan

In the genome of *P. akamusi*, the CYP3 clan consists of 22 genes, which are classified into four families (CYP6, 9, 420, and 3998) and 10 subfamilies. This time, we identified 258 new CYP3 clan proteins from eight other Chironomidae species, and these genes are categorized into four families and eight subfamilies. The CYP6 family is present in all eight species, with varying numbers ranging from 8 to 64. The CYP9 family is found in seven of the eight Chironomidae species, excluding *C. tentans*. However, CYP420 is only present in *C. marinus* and *C. riparius*, while CYP3998 is exclusively found in *B. antarctica*.

The CYP3 clan is the branch with the largest number of genes among the various branches of P450s, primarily composed of members from the CYP6 and CYP9 families. These two families are almost ubiquitous across all insects, with *B. tabaci* being an exception, where its CYP3 clan consists of CYP6, CYP402, and CYP415 [49]. In addition to these family members, most insects also harbor members of the CYP28 family, such as those found in *B. dorsalis*, *C. capitate*, *D. melanogaster*, and *M. domestica* [20,21,22,23]. Apart from the three commonly mentioned families, there are over 30 additional family members in the CYP3 clan, including CYP308–310, CYP317, CYP321, CYP324, CYP329, CYP332, CYP336–CYP338, CYP345–348, and others. The presence and number of these families vary among different species. In Diptera, the CYP3 clan of *D. melanogaster* comprises seven family members. However, in *Ae. aegypti*, *An. gambiae*, and *C. quinquefasciatus*, the CYP3 clan is constituted by only three family members: CYP6, CYP9, and CYP329. In Chironomidae species, the number of families within the CYP3 clan is comparable to that in Culicidae species, consisting of four families: CYP6, CYP9, CYP420, and CYP3998. The quantity of CYP3 clan genes in *C. riparius* and *P. vanderplanki* is on par with that in *Ae. aegypti* and *C. quinquefasciatus*, reaching up to 82 and 51 genes, respectively. In other Chironomidae species, the number of CYP3 clan genes is less than that in *D. melanogaster* and *An. gambiae*, ranging from 15 to 26. Overall, there is a substantial variation in the number of CYP3 clan genes among Chironomidae species, which may be related to their distinct habitats.

**Figure 4:**
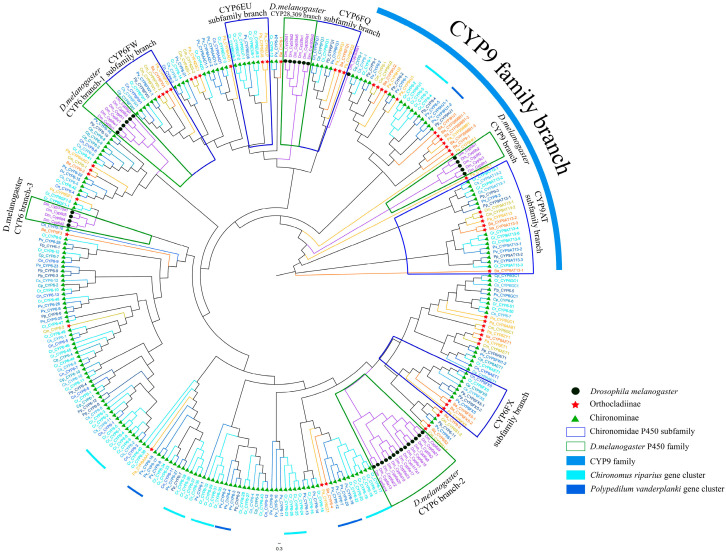
Phylogenetic tree of the CYP3 clan across nine Chironomidae species and *D. melanogaster*, constructed using IQ-TREE (1000 bootstrap replicates). Black circles indicate CYP3 clan members from *D. melanogaster*. Green triangles represent species from the subfamily Chironominae. Red five-pointed stars denote species from the subfamily Orthocladiinae. Major subfamily branches within Chironomidae are outlined with dark blue borders. Different gene family branches of *D. melanogaster* are outlined with green borders. The CYP9 branch is highlighted with a thick blue outer circle. Larger branches for *C. riparius* and *P. vanderplanki* are marked with thin light blue and blue outer circles, respectively.

Unlike the CYP2 clan, the members of the CYP3 clan cannot be clearly distinguished in the phylogenetic tree (Figure 4). Except for CYP308, the CYP3 clan members of *D. melanogaster* are clustered into five single-species sub-branches. Among them, members of the CYP9 family are grouped together. Specifically, members of the CYP9AT subfamily from Chironomidae species cluster into a distinct subfamily branch, which forms a single branch alongside the CYP9 family of *D. melanogaster*. Other CYP9 family members from Chironomidae species cluster with the CYP3998B subfamily to form another branch. This indicates that the CYP9 family emerged early in the evolution of Diptera, and Chironomidae species have undergone species-specific adaptive changes during their evolutionary process. The clustering pattern of the CYP6 family is more complex. The CYP6 members from Chironomidae species cluster into a large branch with other CYP3 clan members of *D. melanogaster*. Most genes within the same subfamily cluster together to form sub-branches, such as the CYP6FQ, CYP6FX, and CYP6EU subfamily branches. These subfamilies likely originated in the common ancestor of Chironomidae and have remained relatively conserved throughout evolution. However, there are instances where genes from different CYP6 subfamilies are grouped within the same sub-branch. This suggests that gene duplication events may have occurred during the later stages of Chironomidae evolution. Additionally, *C. riparius* and *P. vanderplanki* exhibit numerous single-species sub-branches within the CYP6 family. For example, Sub-branch 1 of *C. riparius* contains seven CYP6 family members, while another single-species sub-branch includes four CYP6 family members from *P. vanderplanki*. This indicates that the CYP6 genes in these two Chironomidae species underwent large-scale tandem duplications in the late stages of evolution, generating numerous paralogous gene clusters. Such expansions may be associated with the unique environmental pressures faced by these species. Overall, both the CYP6 and CYP9 families have undergone significant gene duplication events, resulting in the formation of extensive gene clusters. This suggests that Chironomidae species may primarily rely on these two families to cope with diverse and species-specific environmental pressures.

Members of the CYP3 clan play a crucial role in the detoxification of insecticides [17]. The CYP6 and CYP9 families are major members of the CYP3 branch, with a relatively large number of members in each family. Typically, in response to changing and diverse evolutionary pressures, insects generate genes with different functions through gene duplication processes involving these two families, enabling them to adapt to environmental stresses. For example, overexpression of CYP6CM, CYP6ER is associated with high tolerance to imidacloprid in brown planthopper (*Nilaparvata lugens*), *B. tabaci* [59,60]. CYP6B is involved in the metabolism of plant allelochemicals by phytophagous insects, facilitating adaptation to host plants [61,62]. Expression of CYP9J26 enhances tolerance of *Aedes albopictus* to malathion, permethrin, deltamethrin, and propoxur [63]. CYP9A contributes to the detoxification of various pyrethroid insecticides in *Locusta migratoria* [64]. Consequently, “variation” and gene family proliferation within these two families are relatively common in insects [2]. The CYP6 family is unique to insects, and members of both the CYP6 and CYP9 families are associated with the detoxification of xenobiotic substances. A significant proportion of insecticide resistance in insects is attributed to the detoxification functions of these two gene families. For instance, after knocking out the *CYP6HL1*, *CYP6HN1*, and *CYP6HQ1* genes, the mortality rate of the migratory locust, *L. migratoria*, significantly increased following pesticide treatment [2]. The expression of *CYP6FD1* significantly increased in Sogatella furcifera after treatment with flucarbazone-sodium, and the mortality rate significantly increased after gene silencing [65]. The injection of dsLmCYP9A3 increased the mortality rate of migratory locust nymphs exposed to deltamethrin and permethrin [64]. After injecting dsCYP9A40, Spodoptera litura larvae were fed with quercetin, cinnamic acid, deltamethrin, or methoxyfenozide. The results indicated that injecting dsCYP9A40 significantly increased the mortality rate of the larvae, among other effects [66]. There is a notable expansion of members from these two families in chironomid species, especially in *C. riparius* and *P. vanderplanki*, where a large number of gene duplication events have occurred. These expansion events are further corroborated by genomic location data of P450s from these two chironomid species, as we observed a significant clustering of CYP9 and CYP6 members at specific chromosomal loci in both species (Figures S1 and S2). *C. riparius* inhabits organic-rich sediments. Previous studies have shown that when exposed to sediments with the highest levels of contamination by polycyclic aromatic hydrocarbons (PAHs), phthalates, and pesticides, genes related to endocrine pathways, cellular stress responses, and biotransformation processes—including P450 genes—are overexpressed in *C. riparius* [67]. *P. vanderplanki* resides in temporary rock pools in semi-arid regions of Africa and can survive almost complete cellular water loss in a metabolically inactive state [68]. This arid environment induces the expression of certain proteins in *P. vanderplanki* [69]. Therefore, the duplication of CYP6 and CYP9 family genes in these two species likely represents specialized physiological and molecular adaptations for survival in extreme environments.

### 3.5. CYP4 Clan

In the genome of *P. akamusi*, the CYP4 clan comprises 28 genes, which are classified into 10 families (CYP4, 325, 3013, 3987, 3996, 3997, 4030, 4038, 4702, and 4703) and 18 subfamilies. This time, we identified 225 new CYP4 clan proteins from eight other Chironomidae species, and these genes can be categorized into seven families and 14 subfamilies. The CYP4 family is present in all eight species, albeit in relatively small numbers, ranging from 6 to 20.

In insects, the CYP4 clan ranks second only to the CYP3 clan in gene number and is predominantly composed of the CYP4 family. Besides CYP4, the CYP4 clan includes over 20 other family members, such as CYP311, CYP313, CYP312, CYP316, CYP318, CYP325, CYP349–352, CYP3163, etc. In insects, the composition of the CYP4 clan families and their member quantities exhibit remarkable interspecific variation. For instance, the CYP4 clan of the whitefly *B. tabaci* consists solely of the CYP4 family [49], whereas the CYP4 clan of *C. capitate* comprises seven families [22]. In Diptera, the CYP4 clan of *D. melanogaster* comprises six family members, whereas the CYP4 clan of *Ae. aegypti* and *An. gambiae* consists of only two family members: CYP4 and CYP325 [20,25,26]. *C. quinquefasciatus* has three family members in its CYP4 clan: CYP4, CYP325, and CYP326 [22]. In Chironomidae species, the number of families within the CYP4 clan ranges from 3 to 10. Among them, *B. antarctica* has the fewest, with only three families, while *P. akamusi* has the most, with up to 10 families.

**Figure 5:**
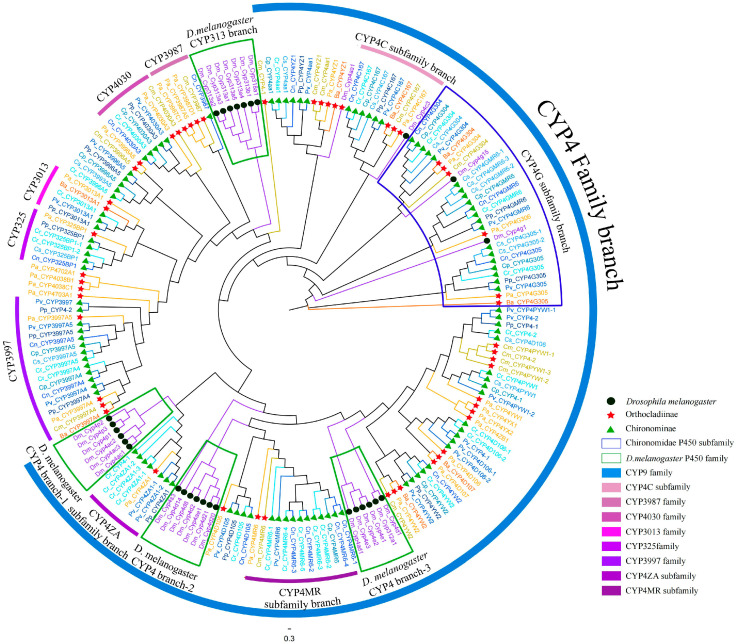
A phylogenetic tree of the CYP3 clan across nine Chironomidae species and *D. melanogaster* was constructed using IQ-TREE (1000 bootstrap replicates), with black circles indicating CYP3 clan members from *D. melanogaster*, green triangles representing Chironominae species, red five-pointed stars denoting Orthocladiinae species, major gene family/subfamily branches within Chironomidae marked by purple outer circles of varying colors, the CYP4G subfamily outlined in blue, different *D. melanogaster* gene family branches outlined in green, and the CYP4 branch highlighted by a thick blue outer circle.

On the phylogenetic tree, members of the CYP4 clan form two major branches, with one large branch being the CYP4 branch (Figure 5). The CYP4 branch exhibits significant gene expansion, where all CYP4 family genes from nine Chironomidae species cluster together with the CYP4 family members of *D. melanogaster*. Among them, the CYP4 family members of *D. melanogaster* aggregate into three single-species clades containing 5, 6, and 7 members, respectively, with Clade 3 also including a member from the CYP312 family. 4G15 and 4G1 branch off independently and cluster with the CYP4G subfamily members from Chironomidae species to form two small CYP4G subfamily clades, which together constitute a larger CYP4G subfamily branch. This suggests that the CYP4G subfamily may have emerged early in evolution, resulting in higher conservation compared to other CYP4 subfamilies. These members likely share functional similarities and have been subjected to similar environmental pressures during evolution. Other CYP4 family genes from Chironomidae species primarily cluster together in the form of subfamilies, such as CYP4MR and CYP4AZ. However, there are also instances where members from different subfamilies are grouped together, indicating that CYP4 family members may have undergone frequent gene duplication events during evolution, which occurred after the divergence of Chironomidae and Drosophilidae. In the other branch, CYP313 and CYP318 from *D. melanogaster* cluster together to form a single-species sub-branch, while other Chironomidae species cluster according to their families. Compared to the CYP4 family branch, this branch has been more conserved during evolution.

The CYP4 family is potentially implicated in a broader spectrum of biosynthetic and metabolic pathways within insects. Multiple members within the CYP4 family possess detoxification functions and are associated with insecticide resistance. For instance, silencing CYP4 genes enhances the susceptibility of *Diaphorina citri* to insecticides [27]. The expression of CYP4G8 from *Helicoverpa armigera* is two-fold up-regulated in a pyrethroid-resistant strain compared to a susceptible strain [70]. Within the CYP4 family, the insect-specific CYP4G subfamily has been the most extensively studied. CYP4G genes exhibit conservation in insect genomes, with at least one member present in every sequenced insect genome [2]. Members of the CYP4G family have diverse functions: besides being associated with insecticide sensitivity, they are also involved in cuticular wax synthesis in insects. *D. melanogaster* CYP4G1 and its ortholog, *M. domestica* CYP4G2, are the first two identified and functionally characterized members of the CYP4G clan [71]. They encode an oxidative decarbonylase that catalyzes the conversion of aldehydes to hydrocarbons (HCs). An increase in HC content enhances the barrier function of cuticle and is also considered one of the reasons for enhanced insecticide resistance [72]. Members of the CYP4 family are also associated with olfactory-related compounds. They may play a role in regulating olfactory receptors, recognizing odor molecules, and facilitating signal transduction [73]. Besides, they may also be involved in the biosynthesis of insect ecdysteroids and the metabolism of JH and their precursors [74,75,76]. P450s require ATP consumption to supply energy during the exercise of their biological functions. Based on this characteristic, we speculate that this phenomenon may reveal a close internal link between the NADH/ATP generation pathway and the hormone synthesis process in insects. From the perspectives of evolution and functional adaptation, this might explain why the expanded P450 gene family can support flexibility in hormone regulation and detoxification functions [77]. Chironomid species exhibit a notable “gene expansion” in the CYP4 family. Members of the CYP4G subfamily cluster with *D. melanogaster* CYP4G1 and CYP4G15, suggesting high evolutionary conservation and potential functional similarities to the *D. melanogaster* CYP4G family.

### 3.6. Mito Clan

Previously, we identified P450s belonging to five families (CYP12, 301, 302, 314, and 315) and seven subfamilies within the Mito clan in the *P. akamusi* genome. In this current study, we identified 71 new Mito clan proteins from eight other Chironomidae species, and these enzymes are categorized into six families and eight subfamilies. The CYP12 family comprises two subfamilies, CYP12AZ and CYP12P, which are consistent with the previously identified CYP12 family members in *P. akamusi*.

Within insects, the Mito clan primarily comprises 11 family members (12, 49, 301, 302, 314, 315, 333, 334, 339, 353). In most insects, the Mito clan is mainly composed of six family members: 12, 49, 301, 302, 314, and 315, as seen in *B. dorsalis*, *C. capitate*, *M. domestica*, and *B. mori*. A minority of insects possess additional family members. For instance, the small white butterfly, *Pieris rapae*, has family members 333, 339, and 428 [78]; *T. castaneum* has 334 and 353; and *Leptinotarsa decemlineata* has 334 and 353 [79]. *B. tabaci* is an exception, as its Mito clan consists of only two family members: 3133 and 315 [49]. The Mito clan of Diptera insects is similar to that of most insects, being composed of the six family members CYP12, CYP49, CYP301, CYP302, CYP314, and CYP315, as seen in species like *D. melanogaster*, *An. gambiae*, and *Ae. aegypti*. Among chironomid species, most have their Mito clan composed of these six family members. However, we did not identify CYP315 in *C. tentans* nor CYP49 in *P. vanderplanki*.

**Figure 6:**
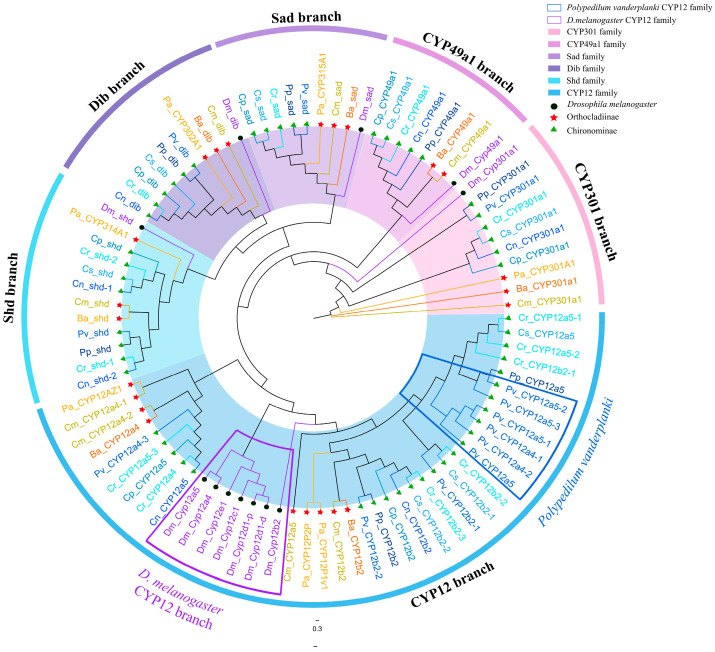
A phylogenetic tree of the Mito clan across nine Chironomidae species and *D. melanogaster* was constructed using IQ-TREE (1000 bootstrap replicates). Black circles indicate *D. melanogaster*, green triangles represent Chironominae species, and red five-pointed stars denote Orthocladiinae species. Different gene families within the Mito clan are marked with outer circles of distinct colors. The CYP12 gene family branch of *P. vanderplanki* is outlined in blue, while the CYP12 gene family branch of *D. melanogaster* is outlined in purple. The abbreviations dib, sad, and shd correspond to *CYP302A1*, *CYP315A1*, and *CYP314A1*, respectively.

The family members of the Mito clan can be clearly distinguished on the phylogenetic tree (Figure 6). Except for the CYP12 family, members of the same family cluster together to form distinct family branches, with each branch containing a homologous gene from *D. melanogaster* and one or two representative genes from various Chironomidae species. This indicates that these families already existed before the divergence of Chironomidae and Drosophilidae, and have been conserved during evolution, without undergoing drastic changes during the evolution of Diptera, possibly related to their conserved functions. The CYP12 family members of *D. melanogaster* cluster into a single-species sub-branch, forming a larger CYP12 branch together with the CYP12 members from Chironomidae species. This suggests that, compared to other Mito clan members, the CYP12 family is less evolutionarily conserved and may have undergone rapid gene duplication events in the later stages of evolution after the divergence of Chironomidae and Drosophilidae. This is consistent with the evolutionary characteristics of Mito clan members observed in other studies. In addition, the six CYP12 genes from *P. vanderplanki* cluster together on the phylogenetic tree, forming a single-species gene cluster. This indicates that gene duplication events occurred in the CYP12 of *P. vanderplanki* during the late stages of evolution, possibly related to the unique environmental stresses it experiences, leading to species-specific adaptive changes.

*CYP302A1* (*dib*), *CYP315A1* (*sad*), and *CYP314A1* (*shd*) within the CYP4 clan participate in the final steps of hydroxylation reactions for the synthesis of insect 20E. Specifically, *CYP302A1* is a carbon-22 hydroxylase that can introduce a hydroxyl group at carbon-22 of 5β-ketotriol, an intermediate in ecdysteroid biosynthesis, and is primarily expressed in the prothoracic gland (PG). CYP315A1 continues to hydroxylate the product catalyzed by *CYP302A1*, 2-deoxyecdysone, thereby generating ecdysone, and finally, *CYP314A1* hydroxylates ecdysone to produce the biologically active 20E [8,80]. When these genes in *Drosophila* undergo mutation, the mutants exhibit embryonic lethality with reduced ecdysteroid titers in embryos. This indicates that these P450 enzymes are crucial for ecdysteroid biosynthesis in *Drosophila* [54,80,81,82,83]. Furthermore, studies in other species have also demonstrated the importance of these three genes in 20E synthesis, such as in Sogatella furcifera, *Laodelphax striatellus* [84]. *Bactrocera minax*, *H. armigera*, *B. mori*, and others [85,86,87]. *CYP301A1* is also conserved across insects. In *D. melanogaster*, Tamar Sztal et al. demonstrated that *CYP301A1* is involved in adult cuticle formation and may participate in ecdysone regulation within the cuticle [88]. There has been relatively limited research on the function of CYP49A1. However, studies on the whole genome of P450 genes in *Ae. aegypti* suggest that CYP49A1 may be involved in electron transfer and steroid biosynthesis. The functions of Mito clan members are highly conserved. Phylogenetic analysis reveals that Mito clan family members in Chironomidae cluster with their homologous proteins in *D. melanogaster* within the same subfamily branches. Thus, they likely share similar functions. Future research should focus on a comprehensive analysis of Mito clan members across Chironomidae species to elucidate their physiological roles.

## 4. Conclusions

The P450 protein family is one of the crucial families in insects, playing significant roles in growth, development, and detoxification. Building upon our previous annotation of the P450 protein family in *P. akamusi*, this study performed de novo annotation and comparative analysis of the P450s in eight additional Chironomidae species, completing a comprehensive phylogenetic analysis across nine Chironomidae species, including *P. akamusi*. We identified a total of 577 P450 proteins, which could be well-classified into four distinct clans in insects. The results indicate that members of the CYP2 clan and the Mito clan in Chironomidae species are highly conserved and exhibit clear orthologous relationships with their Drosophila counterparts. This suggests that, similar to other insects, these two clans in Chironomidae species have maintained a conserved evolutionary pattern. In contrast, members of the CYP3 clan and the CYP4 clan in Chironomidae species have undergone significant and extensive gene duplication events at the species level, with varying degrees of expansion across different species. The two species with the most pronounced expansion are *C. riparius* and *P. vanderplanki*, reflecting differences in the environmental challenges and survival needs faced by different species. Members of these two clans primarily function to cope with rapidly changing environmental pressures.

Our findings, through a comprehensive analysis of the evolutionary relationships of P450 proteins across nine Chironomidae species inhabiting diverse environments, provide new insights into the complex evolutionary processes of insect P450s. The functional studies conducted at different clan levels further elucidate the roles of P450s in Chironomidae species, particularly their relationship with environmental adaptability. This lays a solid foundation for more precise functional characterization and experimental validation in the future. Based on our research, we can screen key P450 genes in Chironomidae species that are associated with pollutant metabolism. By designing environmentally friendly and highly specific RNA interference (RNAi) technologies for chironomid control, we can gain insights into their intrinsic resistance mechanisms. This will facilitate a better explanation and prediction of the adaptive capacities of different chironomid species to the environment, providing a scientific basis for evaluating their potential as environmental indicator organisms. Previous studies have demonstrated that exposure to ordonin can induce the expression of CYP450 enzymes, as evidenced by elevated mRNA and protein levels of key CYP family members. Notably, compounds like ordonin significantly up-regulate CYP3A4 and CYP2C9 expression [89]. Similarly, in chironomid species such as *C. riparius* and *C. tentans*, members of the CYP4, CYP6, and CYP9 gene families exhibit induced expression under stress from various xenobiotic substances [31,32,90]. However, while we have conducted functional predictions for the P450s in these eight Chironomidae species, we have not verified gene expression, inducibility, or conducted validation using transcriptomic data. In the future, we will further validate the functions through experiments such as RNAi, quantitative polymerase chain reaction (QPCR), in vitro expression, and enzyme activity analysis.

## Figures and Tables

**Table 1:** Summary of P450s in nine Chironomidae species.

Clans	Families	*Propsilocerus akamusi*	*Belgica antarctica*	*Clunio* *marinus*	*Chironomus tentans*	*Chironomus tepperi*	*Chironomus riparius*	*Chironomus striatipennis*	*Polypedilum pembai*	*Polypedilum vanderplanki*
2	CYP15	1	0	0	1	1	1	3	1	1
	CYP18	1	1	1	0	1	1	0	1	1
	CYP303	1	1	1	1	1	1	1	1	1
	CYP304	0	0	0	0	0	0	0	2	1
	CYP305	1	0	2	0	0	1	0	1	1
	CYP306	1	1	1	1	1	1	1	1	1
	CYP307	2	2	1	2	2	2	2	1	0
3	CYP3998	1	3	0	0	0	0	0	0	0
	CYP420	1	0	2	0	0	1	0	0	0
	CYP6	15	11	8	18	15	64	16	17	38
	CYP9	5	6	4	0	1	12	3	7	8
	Clan3 (ungrouped)	0	2	1	2	2	5	5	2	5
4	CYP325	1	0	0	1	0	2	1	1	0
	CYP3987	3	0	1	1	0	0	0	0	0
	CYP3996	1	0	1	0	1	1	1	1	1
	CYP3997	2	1	1	2	2	2	1	2	3
	CYP4030	1	0	1	1	1	1	0	1	1
	CYP4038	2	0	0	0	0	0	0	0	0
	CYP4	15	6	11	11	8	20	10	9	16
	CYP4702	1	0	0	0	0	0	0	0	0
	CYP4703	1	0	0	0	0	0	0	0	0
	CYP3013	1	1	0	0	0	1	0	1	1
	Clan4 (ungrouped)	0	1	6	4	4	23	9	7	16
Mito	CYP12	3	2	4	2	2	7	3	2	9
	CYP49	0	1	1	1	1	1	1	1	0
	CYP301	1	1	1	1	1	1	1	1	1
	CYP302	1	1	1	1	1	1	1	1	1
	CYP314	1	1	1	2	1	2	1	1	1
	CYP315	1	1	1	0	1	1	1	1	1
	total	64	43	51	52	47	152	61	63	108

Notes: Different clans are represented by varying shades of green.

## Data Availability

The original contributions presented in this study are included in the Article/Supplementary Materials. Further inquiries can be directed to the corresponding author.

## Supplementary Materials

The following supporting information can be downloaded at: https://www.mdpi.com/article/10.3390/biology14091111/s1, Table S1: Information of genome in nine species; Table S2: The raw data of P450 protein genes in eight non-biting midges. Figure S1: Chromosomal localization of CYP6, CYP9 gene families in the *Polypedilum vanderplanki* genome. Figure S2: Chromosomal localization of CYP6, CYP9 gene families in the *Chironomus riparius* genome.
