# Supplementary material for: Functional Annotation and Comparative Analysis of Cytochrome P450 Protein Family Genes in Nine Chironomidae Species
Source: Biology (Basel). 2025 Aug 22;14(9):1111. doi: 10.3390/biology14091111 (PMC12467003; doi:10.3390/biology14091111)

Figure S1: Chromosomal localization of CYP6 , and CYP9 gene families in the *Polypedilum vanderplanki* genome.

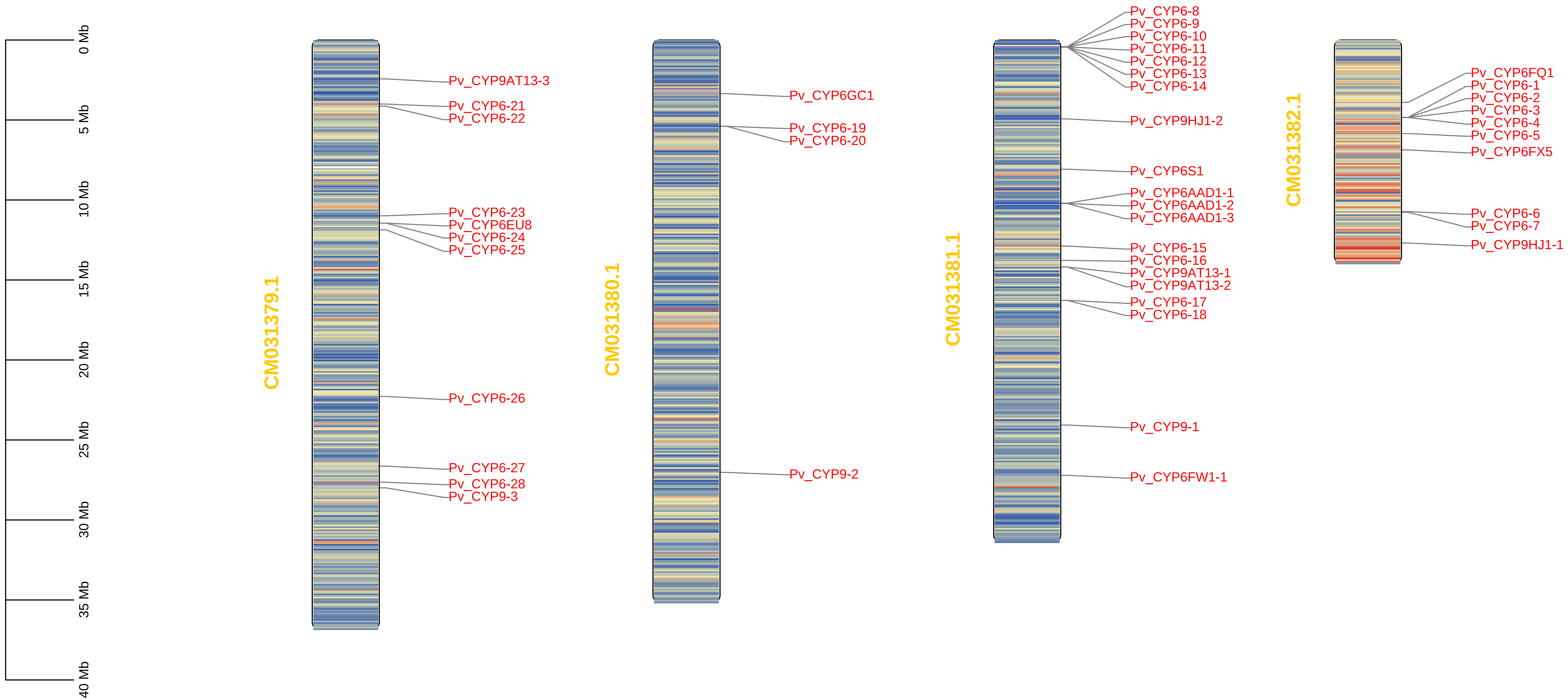

Supplement: Supplementary file 1 [file biology-14-01111-s001.zip › Figure S1.pdf]
