# Supplementary material for: Functional Annotation and Comparative Analysis of Cytochrome P450 Protein Family Genes in Nine Chironomidae Species
Source: Biology (Basel). 2025 Aug 22;14(9):1111. doi: 10.3390/biology14091111 (PMC12467003; doi:10.3390/biology14091111)
Supplement: Supplementary file 1 [file biology-14-01111-s001.zip › Table S1.pdf]

Table S1. Information of genome in nine species.

Species	Genome size	Annotation genes	Publication date	Source
Belgica antarctica	89.6 Mb	13854	Aug 12, 2014	GCA_000775305.1
Clunio marinus	85.5 Mb	21672	Nov 23, 2016	GCA_900005825.1
Chironomus riparius	154.5 Mb	15185	May 31, 2015	GCA_001014505.1
Chironomus striatipennis	181.8 Mb	15586	Oct 23, 2022	GCA_026123125.1
Chironomus tentans	213.5 Mb	15120	Dec 1, 2014	GCA_000786525.1
Chironomus tepperi	202.2 Mb	13987	Jan 18, 2022	GCA_022539635.2
Propsilcerus akamusi	85.83 Mb	11,110	Mar 3, 2021	GCA_018397935.1
Polypedilum pembai	122.9 Mb	15,099	Sep 7, 2020	GCA_014622435.1
Polypedilum vanderplanki	119 Mb	17863	May 10, 2021	GCA_018290095.1
